# Systems mapping of HIV-1 infection

**DOI:** 10.1186/1471-2156-13-91

**Published:** 2012-10-23

**Authors:** Wei Hou, Yihan Sui, Zhong Wang, Yaqun Wang, Ningtao Wang, Jingyuan Liu, Yao Li, Maureen Goodenow, Li Yin, Zuoheng Wang, Rongling Wu

**Affiliations:** 1 Center for Computational Biology, Beijing Forestry University, Beijing, 100081, China; 2 Department of Biostatistics, University of Florida, Gainesville, FL, 32611, USA; 3 Center for Statistical Genetics, Pennsylvania State University, Hershey, PA, 17033, USA; 4 Division of Public Health Sciences, Fred Hutchinson Cancer Research Center, Seattle, WA, 98109, USA; 5 Department of Pathology, Immunology and Laboratory Medicine, University of Florida, Gainesville, FL, 32610, USA; 6 Division of Biostatistics, Yale University, New Haven, CT, 06510, USA

## Abstract

Mathematical models of viral dynamics *in vivo* provide incredible insights into the mechanisms for the nonlinear interaction between virus and host cell populations, the dynamics of viral drug resistance, and the way to eliminate virus infection from individual patients by drug treatment. The integration of these mathematical models with high-throughput genetic and genomic data within a statistical framework will raise a hope for effective treatment of infections with HIV virus through developing potent antiviral drugs based on individual patients’ genetic makeup. In this opinion article, we will show a conceptual model for mapping and dictating a comprehensive picture of genetic control mechanisms for viral dynamics through incorporating a group of differential equations that quantify the emergent properties of a system.

## Introduction

To control HIV-1 virus, antiviral drugs have been developed to prevent the infection of new viral cells or stop already-infected cells from producing infectious virus particles by inhibiting specific viral enzymes [1,2]. Because of the multifactorial complexity of viral-host association, however, the development and delivery of clinically more beneficial novel antiviral drugs have proved a difficult goal [3]. In this essay, we argue that this bottleneck may be overcome by merging two recent advances in mathematical biology and genotyping techniques toward precision medicine. First, viral-drug interactions constitute a complex dynamic system, in which different types of viral cells, including uninfected cells, infected cells, and free virus particles, cooperate with each other and together fight with host immune cells to determine the pattern of viral change in response to drugs [4-6]. A number of sophisticated mathematical models have been developed to describe viral dynamics *in vivo*, providing incredible insights into the mechanisms for the nonlinear interaction between virus and host cell populations, the dynamics of viral drug resistance, and the way to eliminate virus infection from patients by drug treatment [7-15]. Second, the combination between novel instruments and an increasing understanding of molecular genetics has led to the birth of high-throughput genotyping assays such as single nucleotide polymorphisms (SNPs). Through mapping or associating concrete nucleotides or their combinations with the dynamic process of HIV infection [16,17], we can precisely taxonomize this disease by its underlying genomic and molecular causes, thereby enabling the application of precision medicine to diagnose and treat it.

## Systems mapping: a novel tool to dissect complex traits

Beyond a traditional mapping strategy focusing on the static performance of a trait, systems mapping dissolves the phenotype of the trait into its structural, functional or metabolic components through design principles of biological systems, maps the interrelationships and coordination of these components and identifies genes involved in the key pathways that cause the end-point phenotype [18-23]. Systems mapping not only preserves the capacity of functional mapping [24-26] to study the dynamic pattern of genetic control on a time and space scale, but also shows a unique advantage in revealing the dynamic behavior of the genetic correlations among different but developmentally related traits. Its methodological innovation is to integrate mathematical aspects of phenotype formation and progression into a genetic mapping framework to test the interplay between genes and development. Various differential equations which have been instrumental for studying nonlinear and complex dynamics in engineering [27] have shown increasing value and power to quantify the emergent properties of a biological system and interpret experimental results [9-12,28,29].

**Figure 1:**
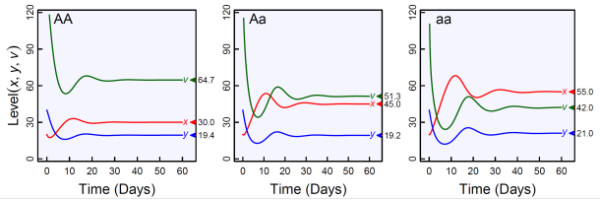
**Numerical simulation showing how a gene affects the dynamics of HIV-1 infection, composed of uninfected cells (*x*), infected cells (*y*), and virus particles (*v*), as described by a basic model (1) in Appendix 1.** The simulated gene has three genotypes *AA*, *Aa* and *aa*, each displaying a different time trajectory for each of these three cell types. Based on these differences, one can test and determine how the gene affects the emerging properties of viral dynamic system, such as average life-times of different cell types and the points of three variables (indicated by triangles) when the system converges to an equilibrium state. The parameter values are (*λ*, *d*, *β*, *a*, *k*, *u*) = (10, 0.01, 0.005, 0.5, 10, 3), (12, 0.01, 0.005, 0.6, 8, 3), and (12, 0.008, 0.005, 0.55, 8, 4) for genotypes *AA*, *Aa* and *aa*, respectively.

The past two decades have witnessed an excellent success in modeling HIV dynamics with differential equations [9-12]. Treating viral-host interactions as a system, Appendix 1 gives a basic model composed of three ordinary differential equations (ODE) for describing the short-term overall dynamics of uninfected cells (*x*), infected cells (*y*), and free virus particles (*v*). These three components together determine the extent and process of pathogenesis according to six ODE parameters, i.e., the rates of production and death of uninfected cells, the rate of production of infected cells from free viruses, the rate of death of infected cells, and the rates of production and death of new viruses from infected cells. Thus, by changing the values of these parameters singly or in combination, the dynamic properties of viral infection, such as viral half life, the limiting ratio of infected to uninfected cells, and the basic reproductive ratio of the virus, can be quantified and predicted [10]. By embedding a system of ODEs within a mixture model framework (Appendix 1), we can use systems mapping to identify specific host genes and their interactions for the pattern of viral dynamics and infection inside a host body. Figure 1 illustrates the characterization of a hypothesized gene that contributes to variation in viral dynamic behavior. Per these genotype-specific changes, an optimal strategy for HIV treatment in terms of the dose and time at which an antiviral drug is administrated can be determined, thus providing a first step toward personalized medicine [23].

**Figure 2:**
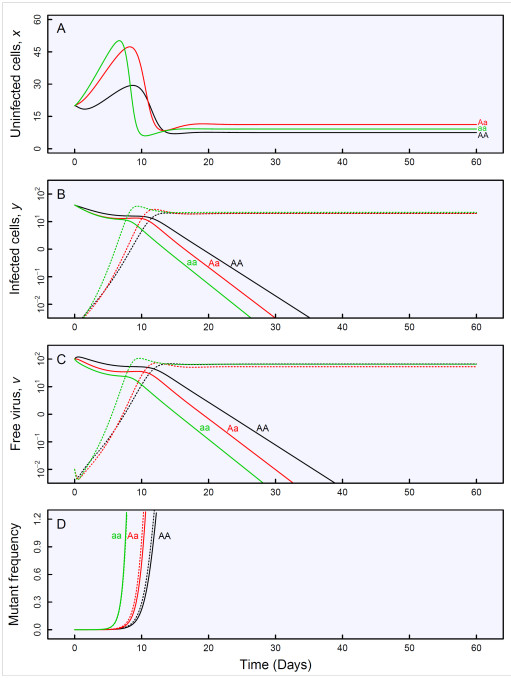
**Simulated genotype-specific differences in the dynamics of drug resistance as described by a model (2) in Appendix 1.** The system simulation focuses on uninfected cell, *x* (**A**), infected cells, *y*, for wild-type virus (solid line) and mutant virus (dash lines) (**B**), and free virus, *v*, for wild-type virus (solid line) and mutant virus (dash line) (**C**), and relative frequency of mutant virus in free virus (solid line) and infected cell population (dash line) (**D**).

In practice, a drug may be resisted if HIV-1 viruses mutate to create new strains [30]. The emergence of drug resistance is a consequence of evolution and presents a response to pressures imposed on the viruses. Different viruses vary in their sensitivity to the drug used and some with greater fitness may be capable of surviving drug treatment [31,32]. In order to understand how viruses are resistant to drugs through mutation, the basic model of Appendix 1 should be expanded to include three additional variables, cells infected by mutant virus, mutant virus particles, and the probability of mutation from wild-type to resistant mutant during reverse transcription of viral RNA into proviral DNA [9]. Systems mapping shows tremendous power to detect genes for virus drug resistance [21] and predict the dynamics of drug resistance (Figure 2). Systems mapping can not only better interpret the genetic mechanisms of drug resistance from experimental data, but also provide scientific guidance on the administration of new antiviral drugs.

## Mapping triple genome interactions

It has been widely accepted that the symptoms and severity of infectious diseases are determined by pathogen-host specificity through cellular, biochemical and signal exchanges [4,33-35]. This specificity, established by undermining a host’s immunological ability to mount an immune response against a particular pathogen, is found to be under genetic determination. Current genetic studies of pathogen-host systems focus on either the host or the pathogen genome, but there is increasing recognition that the complete genetic architecture of pathogen-host specificity, described by the number, position, effect, pleiotropy, and epistasis among genes, involves interactive components from both host and viral genomes [35-38]. In other words, the infection phenotype does not merely result from additive effects of host and pathogen genotypes, but also from specific interactions between the two genomes [35,37].

While many molecular studies define pathogen-host interactions, regardless of the type of hosts, epidemiological models distinguish the difference of hosts as a recipient and transmitter to better characterize the epidemic structure of disease infection, given that infectious diseases like HIV/AIDS are transmitted from an infected person to another [39-41]. From this point of view, the infection outcome should be determined differently but simultaneously by genes from transmitters and recipients. To chart a comprehensive picture of genetic control mechanisms for viral dynamics, we need to address the questions of how genes from viral and host genomes interact to influence viral dynamics and how genetic interactions between recipients and transmitters of virus play a part in the dynamic behavior of viruses. Li et al. [42] pioneered the unification of quantitative genetic theory and epidemiological dynamics for characterizing triple-genome interactions from viruses, transmitters and recipients.

Systems mapping described in Appendix 2 should be embedded within Li et al.’s [42] unifying model to include the interactions of genes derived from the three genomes. This integration allows main genetic effects and epistatic interactions expressed at the genome level to be tested and characterized, including additive effects from the (haploid) viral genome, additive and dominant effects from the transmitter genome, additive and dominant effect from the recipient genome as well as all possible interactions among these main effects. It is interesting to note that the integrated system mapping is capable of estimating and testing high-order epistasis from the viral, recipient and transmitter genomes. Given a growing body of evidence that high-order epistasis is an important determinant of the genetic architecture of complex traits [43-45], systems mapping should be equipped with triple genome interaction modeling.

It should be pointed out that virus evolves through gene recombination and mutations. The genetic machineries that cause viral evolution can be incorporated into systems mapping without technical difficulty. Through such incorporation, systems mapping will provide a useful and timely incentive to detect the genetic control mechanisms of viral dynamics and antivirus drug resistance dynamics and ultimately to design personalized medicine to treat HIV-1 infection from increasingly available genome and HIV data worldwide.

## Toward precision medicine

A major challenge that faces drug development and delivery for controlling viral diseases is to develop computational models for analyzing and predicting the dynamics of decline in virus load during drug therapy and further providing estimates of the rate of emergence of resistant virus. The integration of well-established mathematical models for viral dynamics with high-throughput genetic and genomic data within a statistical framework will raise a hope for effective diagnosis and treatment of infections with HIV virus through developing potent antiviral drugs based on individual patients’ genetic makeup.

In this opinion article, we have provided a synthetic framework for systems mapping of viral dynamics during its progression to AIDS. This framework is equipped with unified mathematical and statistical power to extract genetic information from messy data and possess the analytical and modeling efficiency which does not exist for traditional approaches. By fitting the rate of change of virus infection with clinically meaningful mathematical models, the spatio-temporal pattern of genetic control can be illustrated and predicted over a range of time and space scales. Statistical modeling allows the estimation of mathematical parameters that specify genetic effects on viral dynamics. By genotyping both host and viral genomes, systems mapping is able to identify which viral genes and which human genes from recipients and transmitters determine viral dynamics additively or through non-linear interactions. In this sense, it paves a new way to chart a comprehensive picture of the genetic architecture of viral infection.

An increasing trend in drug development is to integrate it with systems biology aimed to gain deep insights into biological responses. Large-scale gene, protein and metabolite (omics) data that found the building blocks of complex systems have become essential parts of the drug industry to design and deliver new drug [46,47]. However, the true wealth of systems biology will critically rely upon the way of how to incorporate it into human cell and tissue function that affects pathogenesis. By integrating knowledge of organ and system-level responses and omics data, systems mapping will help to prioritize targets and design clinical trials, promising to improve decision making in pharmaceutical development.

## Appendix 1. Mathematical models of viral dynamics

### Basic model

Bonhoeffer et al. [10] developed a basic model for short-term virus dynamics. The model includes three variables: uninfected cells, *x*, infected cells, *y*, and free virus particles, *v*. These three types of cells interact with each other to determine the dynamic changes of virus in a host’s body, which can be described by a system of differential equations:

(1) $$\begin{array}{lll} \overset{\cdot }{x} & = & \lambda -dx-\beta xv\\ \overset{\cdot }{y} & = & \beta xv-ay\\ \overset{\cdot }{v} & = & ky-uv \end{array}$$

where uninfected cells are yielded at a constant rate, *λ*, and die at the rate *dx*; free virus infects uninfected cells to yield infected cells at rate *βxv*; infected cells die at rate *ay*; and new virus is yielded from infected cells at rate *ky* and dies at rate *uv*. The system (1) is defined by six parameters (*λdβaku*) and some initial conditions about *x*, *y*, and *v*.

The dynamic pattern of this system can be determined and predicted by the change of these parameters and the initial conditions of *x*, *y*, and *v*. The basic reproductive ratio of the virus is defined as *R*_0_ = *βλk*/(*adu*). If *R*_0_ is larger than one, then system converges in damped oscillations to the equilibrium x^*^ = *au*/(*βk*), y^*^ = *λ*/*a* – *du*/(*βk*), and v^*^ = *λk*/(*au*) – *d*/*β*. The average life-times of uninfected cells, infected cells, and free virus are given by 1/*d*, 1/*a*, and 1/*u*, respectively. The average number of virus particles produced over the lifetime of a single infected cell (the burst size) is given by *k*/*a*.

### Resistance model

When a treatment is used to control HIV-1, the viruses will produce the resistance to the drug through mutation. The dynamics of drug resistance can be modeled by

(2) $$\begin{array}{lll} \overset{\cdot }{x} & = & \lambda -dx-\beta xv-\beta_{m}xv_{m}\\ \overset{\cdot }{y} & = & \beta \left(1-\varepsilon \right)xv-ay\\ {\overset{\cdot }{y}}_{m} & = & \beta \varepsilon xv+\beta_{m}xv_{m}-ay_{m}\\ \overset{\cdot }{v} & = & ky-uv\\ {\overset{\cdot }{v}}_{m} & = & k_{m}y_{m}-uv_{m} \end{array}$$

where *y*, *y*_*m*_, *v*, and *v*_*m*_ denote cells infected by wild-type virus, cells infected by mutant virus, free wild-type virus, and free mutant virus, respectively [10]. The mutation rate between wild-type and mutant is given by *ε* (in both directions). For a small *ε*, the basic reproductive ratios of wild-type and mutant virus are *R*_0_ = *βλk*/(*adu*) and *R*_0*m*_ = *β*_*m*_*λk*_*m*_/(*adu*).

Model (2) shows that the expected pretreatment frequency of resistant mutant depends on the number of point mutations between wild-type and resistant mutant, the mutation rate of virus replication, and the relative replication rates of wild-type virus, resistant mutant, and all intermediate mutants. Whether or not resistant virus is present in a patient before therapy will crucially depend on the population size of infected cells.

### Cell diversity model

The infected cells may harbor actively replicating virus (*y*_1_), latent virus (*y*_2_) and defective virus (*y*_3_). The basic model (1) can be expanded to include these three types, expressed as

(3) $$\begin{array}{lll} \overset{\cdot }{x} & = & \lambda -dx-\beta xv\\ {\overset{\cdot }{y}}_{w} & = & q_{w}\beta xv-a_{w}y_{w},\;w=1,2,3\\ \overset{\cdot }{v} & = & ky_{1}+cy_{2}-uv \end{array}$$

where *q*_1_, *q*_2_, and *q*_3_ (*q*_1_ + *q*_2_ + *q*_3_ = 1) are the proportions that the cell will immediately enter active viral replication at a rate of virus production k, become latently infected with the virus at a (much slower) rate of virus production c, and produce a defective provirus that will not produce any offspring virus, respectively; and *a*_1_, *a*_2_, and *a*_3_ are the decay rates of actively producing cells, latently infected cells, and defectively infected cells, respectively.

The basic reproductive ratio of the wild-type is *R*_0_ = *βλA*/(*du*). If *R*_0_ is larger than one, then system converges to the equilibrium x^*^ = *u*/(*βA*), $y_{1}^{*}=\frac{q_{1}}{a_{1}}\left(\lambda -\frac{du}{\beta A}\right),y_{2}^{*}=\frac{a_{1}}{a_{2}}\frac{q_{2}}{q_{1}}y_{1}^{*},y_{3}^{*}=\frac{a_{1}}{a_{3}}\frac{q_{3}}{q_{1}}y_{1}^{*}$, and $v^{*}=\frac{\lambda }{u}A-\frac{d}{\beta }$, where $A=\frac{kq_{1}}{a_{1}}+\frac{cq_{2}}{a_{2}}$.

A full model of viral dynamics can be obtained by unifying the resistance model and cell diversity model to form a system of nine ODEs, expressed as

(4) $$\begin{array}{lll} \overset{\cdot }{x} & = & \lambda -dx-\beta xv-\beta_{m}xv_{m}\\ {\overset{\cdot }{y}}_{w} & = & q_{w}\beta \left(1-\varepsilon \right)xv-a_{w}y_{w},\;w=1,2,3\\ {\overset{\cdot }{y}}_{\mathrm{wm}} & = & q_{w}\beta \varepsilon xv+q_{w}\beta_{m}xv_{m}-a_{w}y_{\mathrm{wm}},\;w=1,2,3\\ \overset{\cdot }{v} & = & ky_{1}+cy_{2}-uv\\ {\overset{\cdot }{v}}_{m} & = & k_{m}y_{1m}+c_{m}y_{2m}-uv_{m} \end{array}$$

This group of ODEs provides a comprehensive description of how viral loads change their rate in a time course, how infected cells are generated in response to the emergence of viral particles, and how viral mutation impacts on viral dynamics and drug resistance dynamics. The emerging properties of system (4) were discussed in ref. [10], which can be integrated with systems mapping described in Appendix 2.

## Appendix 2. Systems mapping of viral dynamics

Systems mapping allows the genes and genetic interactions for viral dynamics to be identified by incorporating ODEs into a mapping framework. Consider a segregating population composed of *n* HIV-infected patients genotyped for a set of molecular markers. These patients were repeated sampled to measure uninfected cells (*x*), infected cells (*y*) and viral load (*v*) in their plasma at a series of time points. If specific genes exist to affect the system (1) in Appendix 1, the parameters that specify the system should be different among genotypes. Genetic mapping uses a mixture model-based likelihood to estimate genotype-specific parameters. This likelihood is expressed as

(5) $$L\left(x;y;v\right)=\prod_{i=1}^{n}\left[\omega_{\left(1|i\right)}f_{1}\left(x_{i},y_{i},v_{i}\right)+\ldots +\omega_{\left(J|i\right)}f_{J}\left(x_{i},y_{i},v_{i}\right)\right]$$

where x_*i*_ = (*x*_*i*_(*t*_1_), …, *x*(^*t*^*T*_*i*_)) , y_*i*_ = (*y*_*i*_(*t*_1_), …, *y*(^*t*^*T*_*i*_)) and v_*i*_ = (*v*_*i*_(*t*_1_), …, *v*_*i*_(^*t*^*T*_*i*_)) are the phenotypic values of *x*, *y*, and *v* for subject *i* measured at *T*_*i*_ time points, *ω*_*j|i*_ is the conditional probability of QTL genotype *j* (*j* = 1, …, *J*) given the marker genotype of patient *i*, and *f*_*j*_(x_*i*_,y_*i*_,v_*i*_) is a multivariate normal distribution with expected mean vector for patient *i* that belongs to genotype *j*,

(6) $$\left(m_{xj|i};m_{yj|i};m_{vj|i}\right){}^{\circ}\left(m_{xj|i}\left(t_{1}\right),\ldots ,m_{xj|i}\left({}^{t}T_{i}\right);m_{yj|i}\left(t_{1}\right),\ldots ,m_{yj|i}\left({}^{t}T_{i}\right);m_{vj|i}\left(t_{1}\right),\ldots ,m_{vj|i}\left({}^{t}T_{i}\right)\right)$$

and covariance matrix for subject *i,*

(7) $$\Sigma_{i}=\left(\begin{array}{ccc} \Sigma_{x_{i}} & \Sigma_{x_{i}y_{i}} & \Sigma_{x_{i}v_{i}}\\ \Sigma_{y_{i}x_{i}} & \Sigma_{y_{i}} & \Sigma_{y_{i}v_{i}}\\ \Sigma_{v_{i}x_{i}} & \Sigma_{v_{i}y_{i}} & \Sigma_{v_{i}} \end{array}\right)$$

with $\Sigma_{x_{i}}$, $\Sigma_{y_{i}}$ and $\Sigma_{v_{i}}$ being (*T*_*i*_ × *T*_*i*_) covariance matrices of time-dependent *x*, *y* and *v* values, respectively, and elements off-diagonal being a (*T*_*i*_ × *T*_*i*_) systematical covariance matrix between the two variables.

For a natural population, the conditional probability of functional genotype given a marker genotype (*ω*_*j|i*_) is expressed in terms of the linkage disequilibria between different loci [48]. In systems mapping, we incorporate ODEs (1) of Appendix 1 into mixture model (1) to estimate genotypic means (2) specified by ODE parameters for different genotypes, expressed as (*λ*_*j*_*d*_*j*_*β*_*j*_*a*_*j*_*k*_*j*_*u*_*j*_) for *j* = 1, …, *J*. Since *x*, *y* and *v* variables obey dynamic system (1) of Appendix 1, the derivatives of genotypic means can be expressed in a similar way. Let *g*_*kj|i*_(*tμ*_*kj|i*_) denote the genotypic derivative for variable *k* (*k* = *x*, *y*, or *z*), i.e.,

(8) $$g_{\left(kj|i)(t,\mu_{kj|i}\right)}=\frac{d\mu_{\left(kj|i\right)}}{dt}\text{.}$$

We use *μ*_*kj|i*_ to denote the genotypic mean of variable *j* for individual *i* belonging to genotype *j* at an arbitrary point in a time course. The Runge–Kutta fourth order algorithm can be used to solve the ODEs.

Next, we need to model the covariance structure by using a parsimonious and flexible approach such as an autoregressive, antedependence, autoregressive moving average, or nonparametric and semiparametric approaches. Yap et al. [49] provided a discussion of how to choose a general approach for covariance structure modeling. In likelihood (1), the conditional probabilities of functional genotypes given marker genotypes can be expressed as a function of recombination fractions for an experimental cross population or linkage disequilibria for a natural population [48,50]. The estimation of the recombination fractions or linkage disequilibria can be implemented with the Expectation-Maximization (EM) algorithm.

To demonstrate the usefulness of systems mapping, we assume a sample of *n* HIV-infected patients drawn from a natural human population at random. The sample is analyzed by systems mapping, leading to the detection of a molecular marker which is associated with a QTL that determines the dynamics of drug resistance in a way described by (2) in Appendix 1. At the QTL detected, there are three genotypes *AA*, *Aa* and *aa*, each with a different set of curve parameters (*λ*, *d*, *β*, *β*_*m*_*, a*, *k*, *k*_*m*_, *u, ε*) estimated by systems mapping. We assume that these parameters are estimated as (10, 0.01, 0.005, 0.02, 0.5, 10, 10, 3, 0.0001) for genotype *AA*, (12, 0.01, 0.005, 0.02, 0.6, 8, 8, 3, 0.0001) for genotype *Aa,* and (12, 0.008, 0.005, 0.02, 0.55, 8, 12, 4, 0.0001) for genotype *aa*. Using these estimated values, we draw the curves of drug resistance dynamics for each genotype (Figure 2). Pronounced differences in the form of these curves indicate that the QTL plays an important part in determining the resistance dynamics of drugs used to treat HIV/AIDS.

The model for systems mapping described above can be expanded in two aspects, mathematical and genetic, to better characterize the genetic architecture of viral dynamics. The mathematical expansions are to incorporate the drug resistance model (2), the cell diversity model (3) and the unifying resistance and cell diversity model (4). These expansions allow the functional genes operating at different pathways of viral-host reactions to be identified and mapped, making system mapping more clinically feasible and meaningful. The genetic expansions aim to not only model individual genes from the host or pathogen genome but also characterize epistatic interactions between genes from different genomes. This can be done by expanding the conditional probability of functional genes given marker genotypes *ω*_*j|i*_ using a framework derived by Li et al. [42].

By formulating and testing novel hypotheses, system mapping can address many basic questions. For example, they are

1) How do DNA variants regulate viral dynamics?

2) How do these genes affect the average life-times of uninfected cells, infected cells, and free virus, respectively?

3) How do genes determine the emergence and progression of drug resistance?

4) Are there specific genes that control the possibility of virus eradication by antiviral drug?

5) How important are gene-gene interactions and genome-genome interactions to the dynamic behavior of viral load with or without treatment?

## References

[B1] Smith K Powers KA Kashuba AD Cohen MS HIV-1 treatment as prevention: the good, the bad, and the challenges Curr Opin HIV AIDS 2011 6 4 315 325 21646878 10.1097/COH.0b013e32834788e7 PMC3666589

[B2] Padian NS McCoy SI Karim SSA Hasen N Kim J HIV prevention transformed: the new prevention research agenda Lancet 2011 378 269 278 10.1016/S0140-6736(11)60877-5 21763938 PMC3606928

[B3] Padian NS McCoy SI Balkus JE Wasserheit JN Weighing the gold in the gold standard: challenges in HIV prevention research AIDS 2010 24 621 635 10.1097/QAD.0b013e328337798a 20179575 PMC3695696

[B4] Fellay J Shianna KV Telenti A Goldstein DB Host genetics and HIV-1: The final phase? PLoS Pathog 2010 6 10 e1001033 10.1371/journal.ppat.1001033 20976252 PMC2954832

[B5] Balazs AB Chen J Hong CM Rao DS Yang L Baltimore D Antibody-based protection against HIV infection by vectored immunoprophylaxis Nature 2012 481 81 84 22139420 10.1038/nature10660 PMC3253190

[B6] Sobieszczyk ME Lingappa JR McElrath MJ Host genetic polymorphisms associated with innate immune factors and HIV-1 Curr Opin HIV AIDS 2011 6 427 434 10.1097/COH.0b013e3283497155 21734565

[B7] Ho DD Neumann AU Perelson AS Chen W Leonard JM Rapid turnover of plasma virions and CD4 lymphocytes in HIV-1 infection Nature 1995 373 123 126 10.1038/373123a0 7816094

[B8] Wei X Ghosh SK Taylor ME Johnson VA Emini EA Viral dynamics in human immunodeficiency virus type 1 infection Nature 1995 373 117 122 10.1038/373117a0 7529365

[B9] Perelson AS Neumann AU Markowitz M Leonard JM Ho DD HIV-1 dynamics in vivo: virion clearance rate, infected cell life-span, and viral generation time Science 1996 271 1582 1586 10.1126/science.271.5255.1582 8599114

[B10] Bonhoeffer S May RM Shaw GM Nowak MA Virus dynamics and drug therapy Proc Natl Acad Sci USA 1997 94 6971 6976 10.1073/pnas.94.13.6971 9192676 PMC21269

[B11] Perelson AS Modelling viral and immune system dynamics Nat Rev Immunol 2002 2 28 36 10.1038/nri700 11905835

[B12] Wodarz D Nowak MA Mathematical models of HIV pathogenesis and treatment Bioessays 2002 24 1178 1187 10.1002/bies.10196 12447982

[B13] Simon V Ho DD HIV-1 dynamics in vivo: implications for therapy Nat Rev Microbiol 2003 1 181 190 10.1038/nrmicro772 15035022

[B14] Ribeiro RM Bonhoeffer S Production of resistant HIV mutants during antiretroviral therapy Proc Natl Acad Sci USA 2000 97 7681 7686 10.1073/pnas.97.14.7681 10884399 PMC16603

[B15] Rong L Gilchrist MA Feng Z Perelson AS Modeling within-host HIV-1 dynamics and the evolution of drug resistance: trade-offs between viral enzyme function and drug susceptibility J Theor Biol 2007 247 804 818 10.1016/j.jtbi.2007.04.014 17532343 PMC2265667

[B16] Troyer JL Nelson GW Lautenberger JA Chinn L McIntosh C Genome-wide association study implicates PARD3B-based AIDS restriction J Infect Dis 2011 203 1491 1502 10.1093/infdis/jir046 21502085 PMC3080910

[B17] The International HIV Controllers Study The major genetic determinants of HIV-1 control affect HLA class I peptide presentation Science 2010 330 1551 1557 21051598 10.1126/science.1195271 PMC3235490

[B18] Fu GF Luo J Berg A Wang Z Li JH A dynamic model for functional mapping of biological rhythms J Biol Dyn 2010 4 1 10 10.1080/17513750903332652 21278847 PMC3027063

[B19] Fu GF Wang Z Li JH Wu RL A mathematical framework for functional mapping of complex systems using delay differential equations J Theor Biol 2011 289 206 216 21871898 10.1016/j.jtbi.2011.08.002

[B20] Luo JT Hager WW Wu RL A differential equation model for functional mapping of a virus-cell dynamic system J Math Biol 2010 65 1 15 19685244 10.1007/s00285-009-0288-1

[B21] Guo YQ Luo JT Wang JX Wu RL How to compute which genes control drug resistance dynamics Drug Discov Today 2011 16 334 339 10.1016/j.drudis.2011.02.004 21315181

[B22] Wu RL Cao JG Huang ZW Wang Z Gai JY Systems mapping: How to improve the genetic mapping of complex traits through design principles of biological systems BMC Syst Biol 2011 5 84 10.1186/1752-0509-5-84 21615967 PMC3127792

[B23] Ahn K Luo J Keefe D Wu RL Functional mapping of drug response with pharmacodynamic-pharmcokinetic principles Trend Pharmacolog Sci 2010 31 306 311 10.1016/j.tips.2010.04.004 20488563

[B24] Ma CX Casella G Wu RL Functional mapping of quantitative trait loci underlying the character process: a theoretical framework Genetics 2002 161 1751 1762 12196415 10.1093/genetics/161.4.1751 PMC1462199

[B25] Wu RL Lin M Functional mapping – How to map and study the genetic architecture of dynamic complex traits Nat Rev Genet 2006 7 229 237 16485021 10.1038/nrg1804

[B26] Li Y Wu RL Functional mapping of growth and development Biol Rev 2010 85 207 216 19930171 10.1111/j.1469-185X.2009.00096.x

[B27] Beretta E Kuang Y Geometric stability switch criteria in delay differential systems with delay dependent parameters SIAM J Math Anal 2002 33 1144 1165 10.1137/S0036141000376086

[B28] Barabasi AL Oltvai ZN Network biology: understanding the cell’s functional organization Nat Rev Genet 2004 5 101 113 10.1038/nrg1272 14735121

[B29] Boone C Bussey H Andrews BJ Exploring genetic interactions and networks with yeast Nat Rev Genet 2007 8 437 449 10.1038/nrg2085 17510664

[B30] McKeegan KS Borges-Walmsley MI Walmsley AR Microbial and viral drug resistance mechanisms Trends Microbiol 2002 10 s8 s14 10.1016/S0966-842X(02)02429-0 12377562

[B31] Davies J Davies D Origins and evolution of antibiotic resistance Microbiol Mol Biol Rev 2010 74 417 433 10.1128/MMBR.00016-10 20805405 PMC2937522

[B32] Toprak E Veres A Michel JB Chait R Hartl DL Kishony R Evolutionary paths to antibiotic resistance under dynamically sustained drug selection Nat Genet 2011 44 101 105 10.1038/ng.1034 22179135 PMC3534735

[B33] Thompson JN Burdon JJ Gene-for-gene coevolution between plants and parasites Nature 1992 360 121 126 10.1038/360121a0

[B34] Tetard-Jones C Kertesz MA Gallois P Preziosi RF Genotype-by-genotype interactions modified by a third species in a plantinsect system Am Nat 2007 170 492 499 10.1086/520115 17879200

[B35] Lambrechts L Dissecting the genetic architecture of host–pathogen specificity PLoS Pathog 2010 6 8 e1001019 10.1371/journal.ppat.1001019 20700450 PMC2916863

[B36] Persson J Vance RE Genetics-squared: combining host and pathogen genetics in the analysis of innate immunity and bacterial virulence Immunogenetics 2007 59 761 778 10.1007/s00251-007-0248-0 17874090

[B37] Wang Z Hou W Wu R A statistical model to analyse quantitative trait locus interactions for HIV dynamics from the virus and human genomes Stat Med 2006 25 495 511 10.1002/sim.2219 16013038

[B38] Martinez J Fleury F Varaldi J Heritable variation in an extended phenotype: the case of a parasitoid manipulated by a virus J Evol Biol 2012 25 54 65 10.1111/j.1420-9101.2011.02405.x 22023097

[B39] Galvin SR Cohen MS The role of sexually transmitted diseases in HIV transmission Nat Rev Microbiol 2004 2 33 42 10.1038/nrmicro794 15035007

[B40] Coombs RW Reichelderfer PS Landay AL Recent observations on HIV type-1 infection in the genital tract of men and women AIDS 2003 17 455 480 10.1097/00002030-200303070-00001 12598766

[B41] Gupta K Klasse PJ How do viral and host factors modulate the sexual transmission of HIV? Can transmission be blocked? PLoS Med 2006 3 2 e79 10.1371/journal.pmed.0030079 16492075 PMC1388058

[B42] Li Y Berg A Chang MN Du P Ahn K A statistical model for genetic mapping of viral infection by integrating epidemiological behavior Stat Appl Genet Mol Biol 2009 8 1 38 10.2202/1544-6115.1475 PMC2861318 19799557

[B43] Wang Z Liu T Lin ZW Hegarty J Koltun WA A general model for multilocus epistatic interactions in case–control studies PLoS One 2010 5 8 e11384 10.1371/journal.pone.0011384 20814428 PMC2909900

[B44] Pettersson M Besnier F Siegel PB Carlborg Ö Replication and explorations of high-order epistasis using a large advanced intercross line pedigree PLoS Genet 2011 7 7 e1002180 10.1371/journal.pgen.1002180 21814519 PMC3140984

[B45] Imielinski M Belta C Exploiting the pathway structure of metabolism to reveal high-order epistasis BMC Syst Biol 2008 2 40 10.1186/1752-0509-2-40 18447928 PMC2390508

[B46] Butcher EC Berg EL Kunkel EJ Systems biology in drug discovery Nat Biotech 2004 22 1253 1259 10.1038/nbt1017 15470465

[B47] Hopkins AL Network pharmacology: the next paradigm in drug discovery Nat Chem Biol 2008 4 682 690 10.1038/nchembio.118 18936753

[B48] Wu RL Zeng ZB Joint linkage and linkage disequilibrium mapping in natural populations Genetics 2001 157 899 909 11157006 10.1093/genetics/157.2.899 PMC1461502

[B49] Yap J Fan JWRL Nonparametric modeling of covariance structure in functional mapping of quantitative trait loci Biometrics 2009 65 1068 1077 10.1111/j.1541-0420.2009.01222.x 19302406 PMC2987658

[B50] Wu RL Ma CX Casella G Statistical Genetics of Quantitative Traits: Linkage, Maps, and QTL 2007 New York: Springer

